# Supplementary material for: AKAP79 enables calcineurin to directly suppress protein kinase A activity
Source: eLife. 2021 Oct 6;10:e68164. doi: 10.7554/eLife.68164 (PMC8560092; doi:10.7554/eLife.68164)
Supplement: Supplementary file 1. — Parameter terminology is according to the numbers above stated in Figure 4D, for example k12 refers to the on rate of cAMP binding to state 1 (pRII-C) to produce state 2 (pRII-C-cAMP). The prior range used to constrain parameter estimation is provided for each parameter along with links to the references used to set the default values. [file elife-68164-supp1.docx]

**Supplementary File 1**

| **Parameter** | | **Default Value** | **Parameter Range for ABC** | | **Reference** |
| --- | --- | --- | --- | --- | --- |
| ***Parameters for phosphorylated RII subunits*** | | | | | |
| k12 (pRII-C + cAMP → pRII-C-cAMP) | | 0.496 μM^-1^ s^-1^ | 4.96x10^-4^ to 4.96x10^2^ μM^-1^ s^-1^ | | (Isensee et al., 2018) |
| k21 (pRII-C + cAMP ← pRII-C-cAMP) | | 0.347 s^-1^ | k21=k12xK_D_12 | |  |
| k32 (pRII-cAMP + C → pRII-C-cAMP) | | 5.45x10^-3^ μM^-1^ s^-1^ | 5.45x10^-6^ to 5.45 μM^-1^ s^-1^ | | (Zhang et al., 2012) |
| k23 (pRII-cAMP + C ← pRII-C-cAMP) | | 1.56x10^-2^ s^-1^ | 1.56x10^-5^ to 1.56x10^1^ s^-1^ | |  |
| k43 (pRII + cAMP → pRII-cAMP) | | 0.015 μM^-1^ s^-1^ | 1.5x10^-5^ to 1.5x10^1^ μM^-1^ s^-1^ | | Ogreid & Doskeland, 1981) (Isensee et al., 2018) |
| k34 (pRII + cAMP ← pRII-cAMP) | | 1.60x10^-3^ s^-1^ | 1.60x10^-6^ to 1.60 s^-1^ | |  |
| k41 (pRII + C → pRII-C) | | 3.8x10^-2^ μM^-1^ s^-1^ | 3.80x10^-5^ to 3.8x10^1^ μM^-1^ s^-1^ | | (Zhang et al., 2015) |
| k14 (pRII + C ← pRII-C) | | 2.60x10^-3^ s^-1^ | 2.60x10^-6^ to 2.60 s^-1^ | |  |
| K_D_12 | | 0.7 μM | 0.35 to 1.4 μM | | (Zhang et al., 2012) |
| ***Parameters for dephosphorylated RII subunits*** | | | | | |
| k56 (RII-C + cAMP → RII-C-cAMP) | | 0.496 μM^-1^ s^-1^ | 4.96x10^-4^ to 4.96x10^2^ μM^-1^ s^-1^ | | (Isensee et al., 2018) |
| k65 (RII-C + cAMP ← RII-C-cAMP) | | 1.43 s^-1^ | 1.43x10^-3^ to 1.43x10^3^ s^-1^ | |  |
| k76 (RII-cAMP + C → RII-C-cAMP) | | 2.98x10^-1^ μM^-1^ s^-1^ | 2.98x10^-4^ to 2.984x10^2^ μM^-1^ s^-1^ | | (Zhang et al., 2015; Zhang et al., 2012) |
| k67 (RII-cAMP + C ← RII-C-cAMP) | | 1.80x10^-2^ s^-1^ | 1.80x10^-5^ to 1.80x10^1^ s^-1^ | |  |
| k87 (RII + cAMP → RII-cAMP) | | 0.015 μM^-1^ s^-1^ | 1.5x10^-5^ to 1.5x10^1^ μM^-1^ s^-1^ | | (Ogreid & Doskeland, 1981) |
| k78 (RII + cAMP ← RII-cAMP) | | 1.60x10^-3^ s^-1^ | 1.60x10^-6^ to 1.60 s^-1^ | |  |
| k85 (RII + C → RII-C) | | 2.10 μM^-1^ s^-1^ | 2.10x10^-3^ to 2.10x10^3^ μM^-1^ s^-1^ | | (Zhang et al., 2015) |
| k58 (RII + C ← RII-C) | | 3.0x10^-4^ s^-1^ | 3.0x10^-7^ to 3.0x10^-1^ s^-1^ | |  |
| ***Parameters for phosphorylation of RII subunits*** | | | | | |
| k51 (RII-C → pRII-C) | | 33 s^-1^ | 3.30x10^-2^ to 3.30x10^4^ s^-1^ | | (Moore et al., 2003) |
| k62 (RII-C-cAMP → pRII-C-cAMP) | | 33 s^-1^ | 3.30x10^-2^ to 3.30x10^4^ s^-1^ | | Equal to k51 |
| ***Parameters for dephosphorylation of RII subunits*** | | | | | |
| k44’ (pRII + CN → pRII-CaN) | | 0.226 μM^-1^ s^-1^ | k44'=(k4'4+k4'8)/K_Mwithout_ | | (Stemmer & Klee, 1994) |
| k4’4 (pRII + CN ← pRII-CN) | | 20 s^-1^ | 2.0x10^-2^ to 2.0x10^4^ s^-1^ | |  |
| k33’ (pRII-cAMP + CN → pRII-cAMP-CN) | | 0.226 μM^-1^ s^-1^ | k33'=(k3'3+k3'7)/K_Mwithout_ | | Equal to k44’ |
| k3’3 (pRII-cAMP + CN ← pRII-cAMP-CN) | | 20 s^-1^ | 2.0x10^-2^ to 2.0x10^4^ s^-1^ | | Equal to k4’4 |
| k4’8 (pRII-CN → RII + CN) | | 2.6 s^-1^ | 2.6x10^-3^ to 2.6x10^3^ s^-1^ | | (Stemmer & Klee, 1994) |
| k3’7 (pRII-cAMP-CN → RII-cAMP + CN) | | 2.6 s^-1^ | 2.6x10^-3^ to 2.6x10^3^ s^-1^ | | Equal to k4’8 |
| K_Mwithout_ (without AKAP79) | | 100 μM | 66.7 to 150 μM | | This study; Stemmer & Klee, 1994) |
| ***Parameters for dephosphorylation of RII subunits when AKAP79 is present*** | | | | | |
| k44’-AKAP (pRII + CN → pRII-CN) | | 2.33 μM^-1^ s^-1^ | k44'-AKAP=(k4'4-AKAP+k4'8-AKAP)/K_Mwith_ | | This study |
| k4’4-AKAP (pRII + CN ← pRII-CN) | | 2 s^-1^ | 2.0x10^-3^ to 2.0x10^3^ s^-1^ | |  |
| k33’-AKAP (pRII-cAMP + CN → pRII-cAMP-CN) | | 2.33 μM^-1^ s^-1^ | k33'-AKAP=(k3'3-AKAP+k3'7-AKAP)/K_Mwith_ | | Equal to k44’-AKAP |
| k3’3-AKAP (pRII-cAMP + CN ← pRII-cAMP-CN) | | 2 s^-1^ | 2.0x10^-3^ to 2.0x10^3^ s^-1^ | | Equal to k4’4-AKAP |
| k4’8-AKAP (pRII-CN → RII + CN) | | 0.45 s^-1^ | 0.237 to 0.855 s^-1^ | | This study |
| k3’7-AKAP ((pRII-cAMP-CN → RII-cAMP + CN) | | 0.45 s^-1^ | 0.237 to 0.855 s^-1^ | | Equal to k4’8-AKAP |
| K_Mwith_ (when AKAP79 is present) | | 1 μM | 0.67 to 1.5 μM | | This study |
| ***Parameters for AKAR4 phosphorylation*** | | | | | |
| AKAR4 + C → AKAR4-C | | 1.82x10^-2^ μM^-1^ s^-1^ | Fixed | | This study |
| AKAR4 + C ← AKAR4-C | | 0.106 s^-1^ | Fixed | |  |
| AKAR4-C → pAKAR4 + C | | 10.2 s^-1^ | Fixed | |  |
| ***Initial conditions for molecules*** | | | | | |
| **Molecules** | **Concentration** | | | **Reference** | |
| RII | 6.30 μM | | | This study | |
| RII_C | 0.63 μM | | |  |  |
| CN | 0 or 1.5 μM | | |  |  |
| cAMP | 0, 0.2, 1 and 2 μM | | |  |  |
