# Supplementary material for: AKAP79 enables calcineurin to directly suppress protein kinase A activity
Source: eLife. 2021 Oct 6;10:e68164. doi: 10.7554/eLife.68164 (PMC8560092; doi:10.7554/eLife.68164)
Supplement: Supplementary file 2. [file elife-68164-supp2.docx]

**Supplementary File 2**

| **Primer Name** | **Sequence (5’ to 3’)** |
| --- | --- |
| EcoI_8HisNLS_XbaI | AATTCGCCGGCCACCACCACCACCACCACCACCACGGCGCCCTGCCCCCCCTGGAGCGCCTGACCCTGTAAT |
| XbaI_8HisNLS_EcoRI | ctagattacagggtcaggcgctccagggggggcagGGCGCCGTGGTGGTGGTGGTGGTGGTGGTGGCCGGCG |
| XbaI_8HisNLS_EcoRI | ctagattacagggtcaggcgctccagggggggcagGGCGCCGTGGTGGTGGTGGTGGTGGTGGTGGCCGGCG |
| Nde1_AKAP79_331 | CATGGCAGCCATATGCATCATCACCATCATCATAAAAGAATGGAGCCAATTG |
| Nde1_AKAP79_1 | CATGGCAGCCATATGCATCATCACCATCATCATATGAGCCACATCCAGATCC |
| AKAP79_427_EcoRI | ACAGAATTCTCACTGTAGAAGATTGTTTATTTTATTATCATCAGAG |
| hS98A_F | GATTTAATAGACGAGTAGCAGTCTGTGCTGAGACC |
| hS98A_R | GGTCTCAGCACAGACTGCTACTCGTCTATTAAATC |
| hS98E_F | CTAGCAGATTTAATAGACGAGTAGAAGTCTGTGCTGAGACCTATAACCCTG |
| hS98E_R | CAGGGTTATAGGTCTCAGCACAGACTTCTACTCGTCTATTAAATCTGCTAG |
| ΔPKA_F | GAGGATAGAACTTCAGAACAATATGAAACAAAGAATGCTATTCAGTTGTCAATAGAAC |
| ΔPKA_R | GTTCTATTGACAACTGAATAGCATTCTTTGTTTCATATTGTTCTGAAGTTCTATCCTC |
| Prkar2a_F | TAGAATTCCACATGAGCCACATCCAGATCCCAC |
| Prkar2a_R | TAGGATCCGAGCTACTGCCCGGGGTCCAATAGATC |
| Prkar2a_shRNA_resist_F | GCGAGGCCCGCCGGCAAGAGTCAGACTCGTTCA |
| Prkar2a_shRNA_resist_R | TGAACGAGTCTGACTCTTGCCGGCGGGCCTCGC |
| shRIIα_F1 | GCCAGGAATCAGACTCGTTCATTCAAGAGATGAACGAGTCTGATTCCTGGCTTTTTTGT |
| shRIIα_R1 | CTAGACAAAAAAGCCAGGAATCAGACTCGTTCATCTCTTGAATGAACGAGTCTGATTCCTGGC |
| shRIIα_2F | GGAAGCCTGTAAAGACATTTTCAAGAGAAATGTCTTTACAGGCTTCCTTTTTTGT |
| shRIIα_2R | CTAGACAAAAAAGGAAGCCTGTAAAGACATTTCTCTTGAAAATGTCTTTACAGGCTTCC |
| shRIIα_3F | GGCAGTAGATGTGATGAATTTCAAGAGAATTCATCACATCTACTGCCTTTTTTGT |
| shRIIα_3R | CTAGACAAAAAAGGCAGTAGATGTGATGAATTCTCTTGAAATTCATCACATCTACTGCC |
| shScram_F | GCCACGTCATAGAGACACTGTTTCAAGAGAACAGTGTCTCTATGACGTGGCTTTTTTGT |
| shScram_R | CTAGACAAAAAAGCCACGTCATAGAGACACTGTTCTCTTGAAACAGTGTCTCTATGACGTGGC |
| rS97A_F | CCAGCAAATTTACTAGACGAGTAGCAGTCTGTGCAGAAAC |
| rS97A_R | GTTTCTGCACAGACTGCTACTCGTCTAGTAAATTTGCTGG |
| rS97E_F | CCGATTCCCAGCAAATTTACTAGACGAGTAGATGTCTGTGCAGAAACGTT |
| rS97E_R | AACGTTTCTGCACAGACATCTACTCGTCTAGTAAATTTGCTGGGAATCGG |
| RIIα-IRES-EGFP_F | GACGAGCTGTACAAGTAAACCGGTGCGGCCGCGACTCTAGATCATAATCAGCCATACCACATTTGTAGA |
| RIIα-IRES-EGFP_R | TCTACAAATGTGGTATGGCTGATTATGATCTAGAGTCGCGGCCGCTGGCCATTTACTTGTACAGCTCGTC |
| FUGW_NheI_F | GGCTGCTGGGCTAGCCGGGGCTTTC |
| FUGW_NheI_R | GAAAGCCCCGGCTAGCCCAGCAGCC |
