# Supplementary figures and images for: AKAP79 enables calcineurin to directly suppress protein kinase A activity
Source: eLife. 2021 Oct 6;10:e68164. doi: 10.7554/eLife.68164 (PMC8560092; doi:10.7554/eLife.68164)

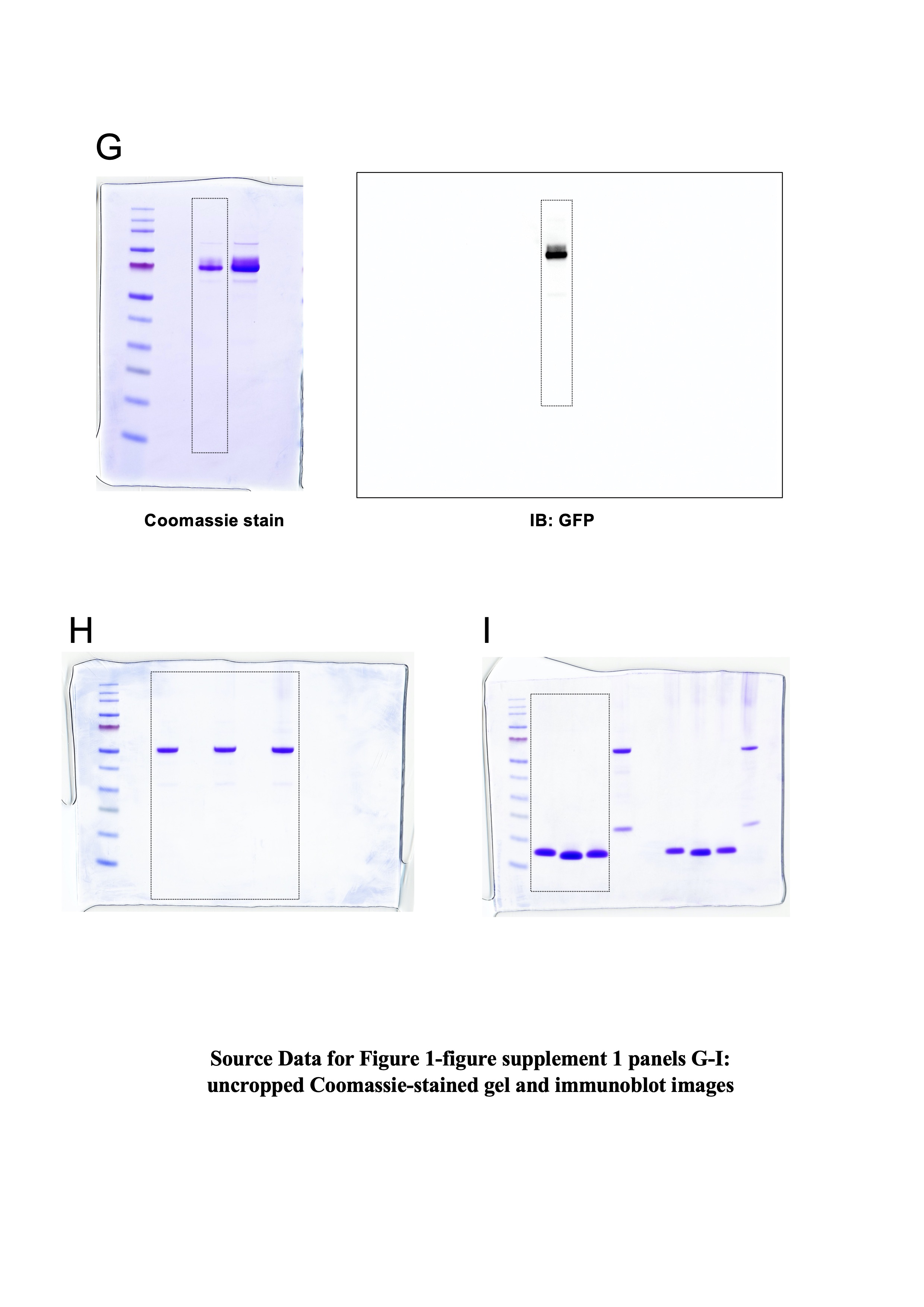

Supplement: Source data 1. [file elife-68164-supp3.zip › Fig1_Sup1_PanelsGtoI_Uncropped_Images.jpg]

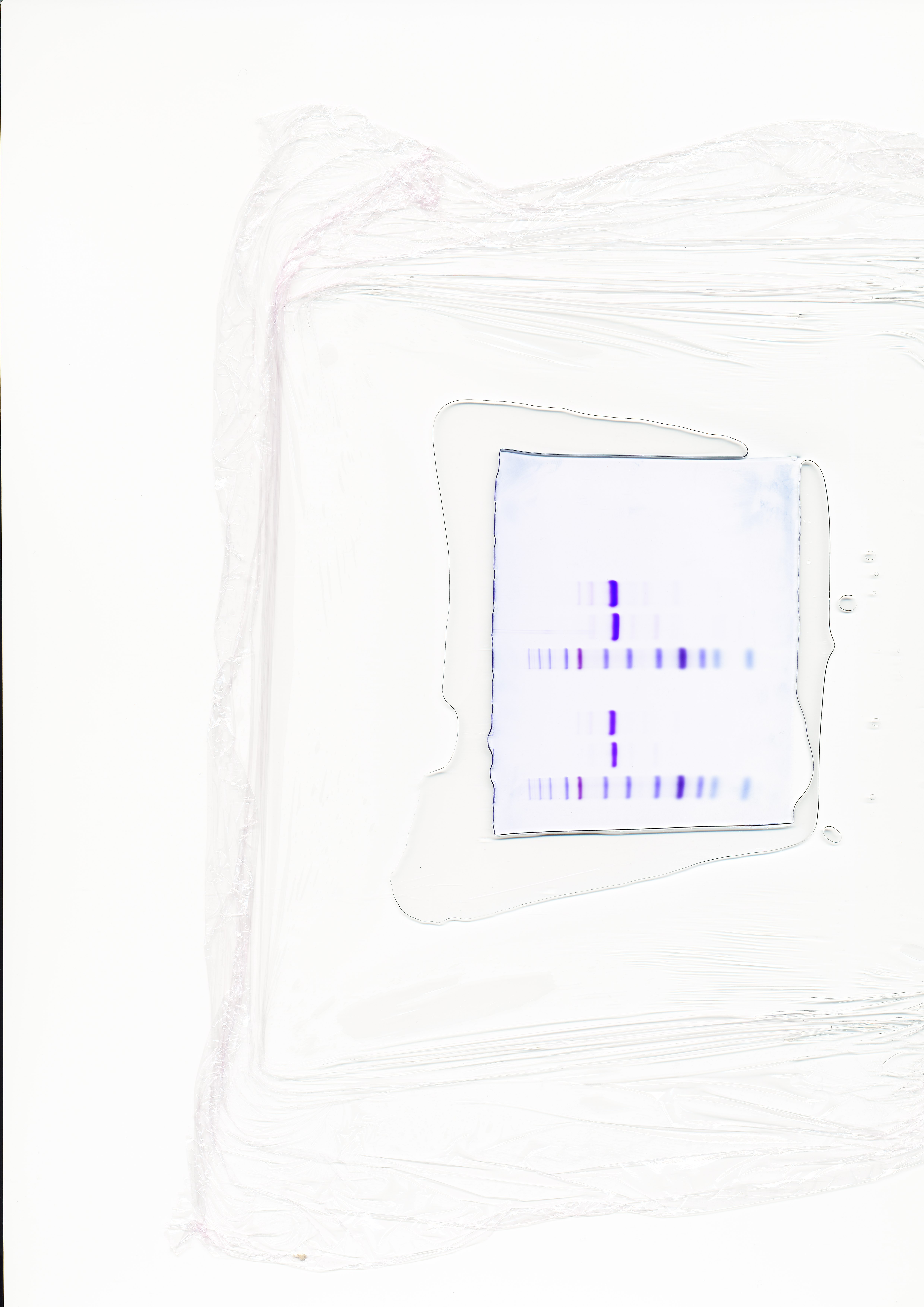

Supplement: Source data 1. [file elife-68164-supp3.zip › Fig1_Sup1A_Coomassie_Original_Image.jpg]

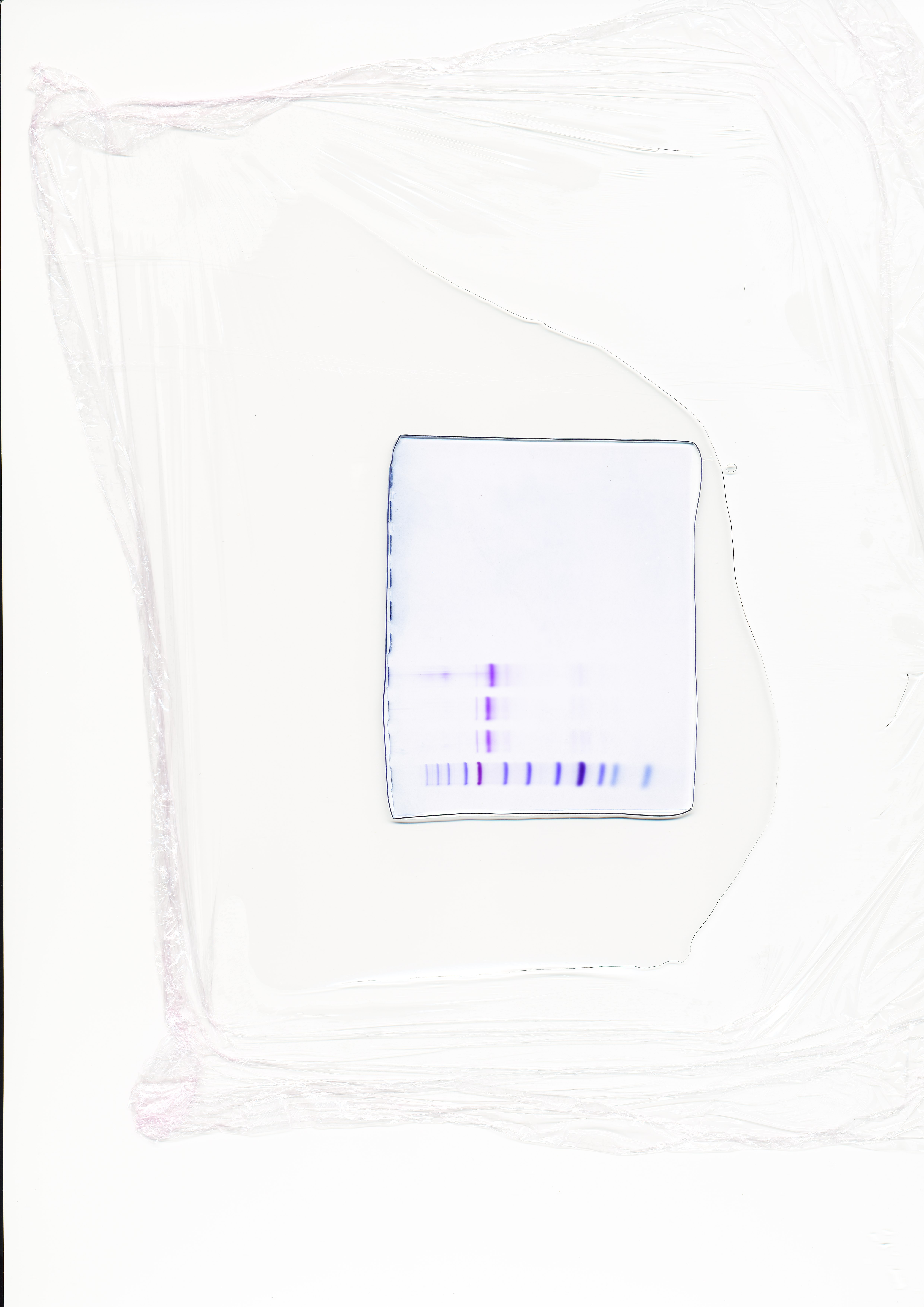

Supplement: Source data 1. [file elife-68164-supp3.zip › Fig1_Sup1B_Coomassie_Original_Image.jpg]

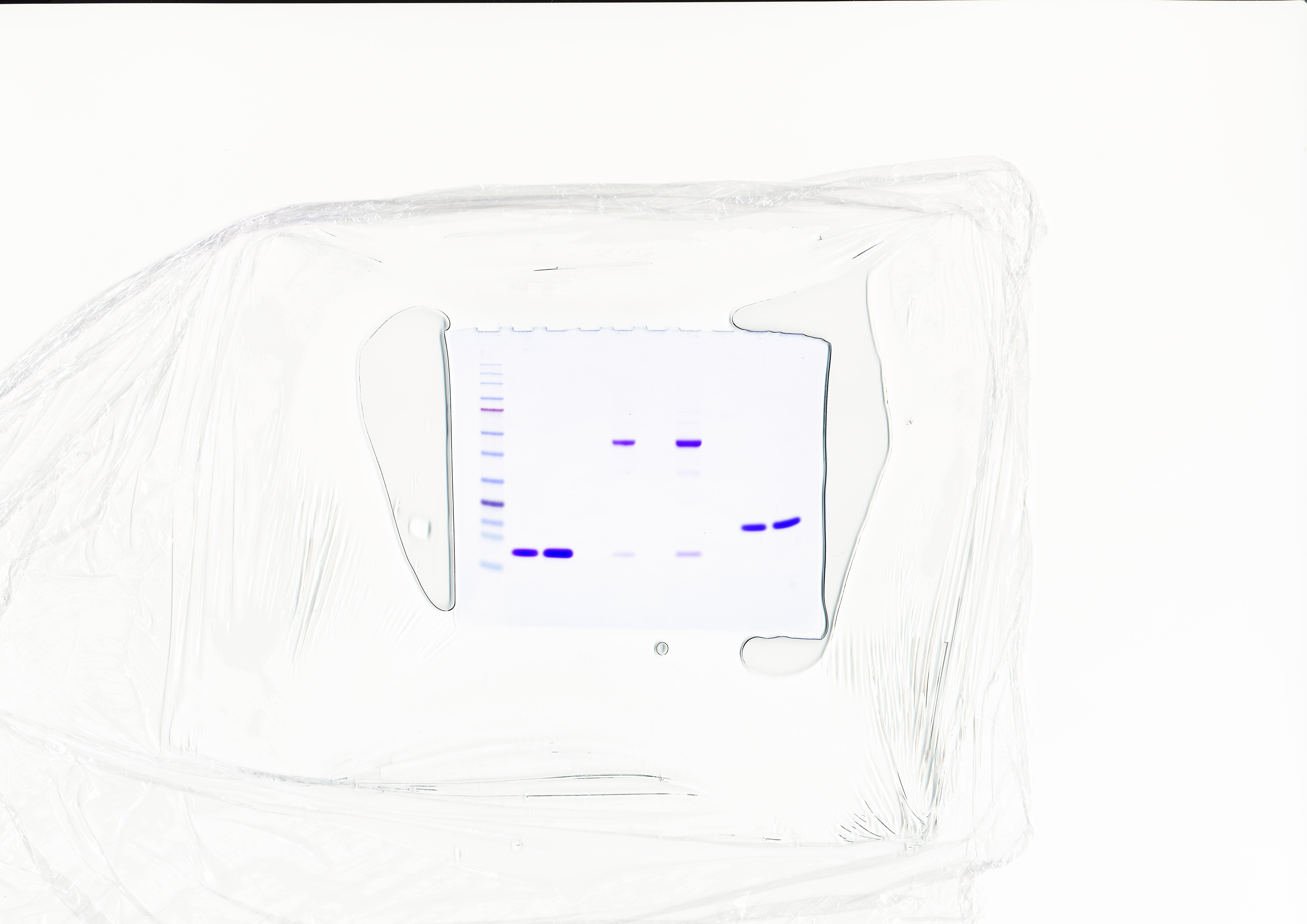

Supplement: Source data 1. [file elife-68164-supp3.zip › Fig1_Sup1Cand1F_Coomassie_Original_Image.jpg]

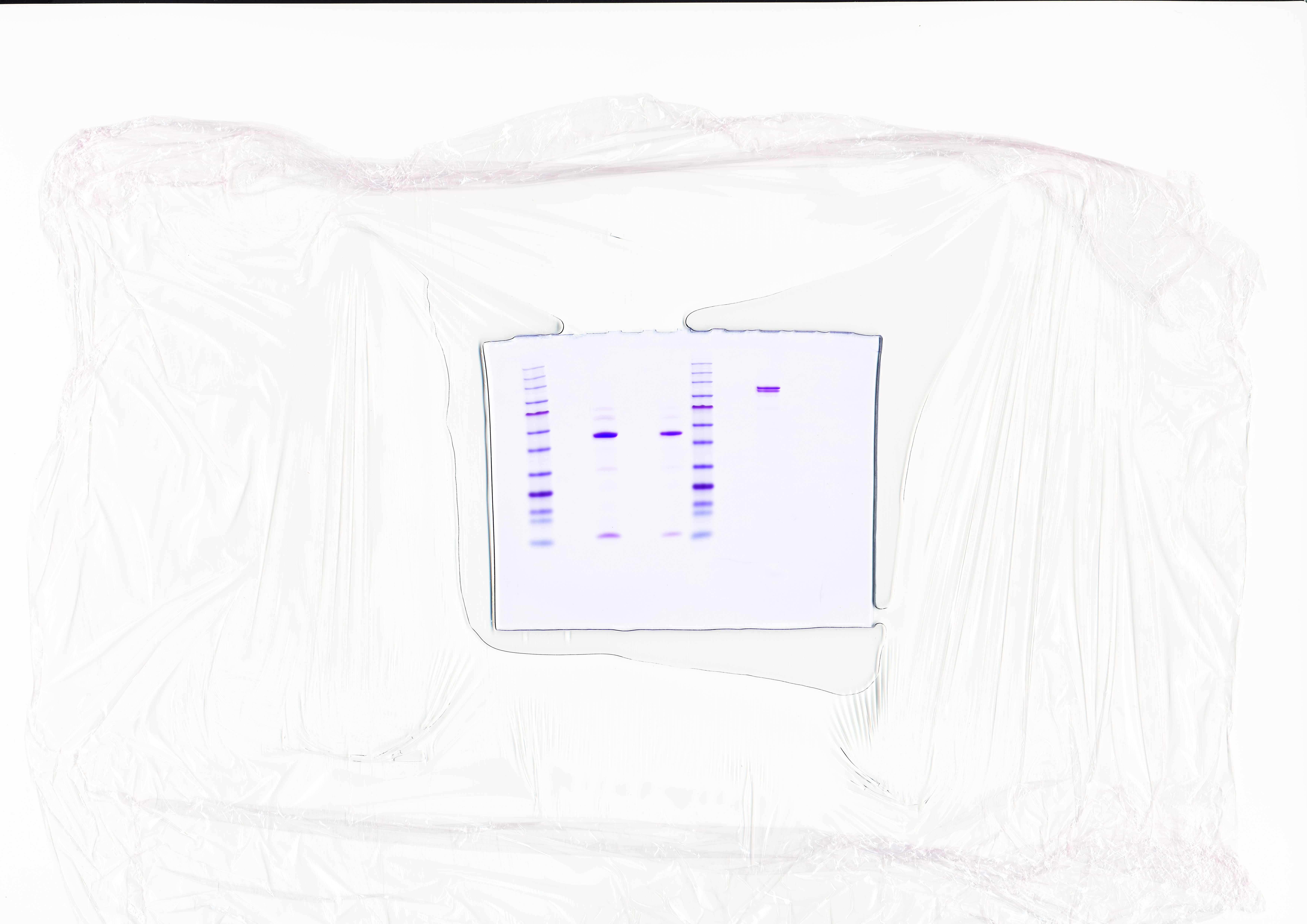

Supplement: Source data 1. [file elife-68164-supp3.zip › Fig1_Sup1D_Coomassie_Image.jpg]

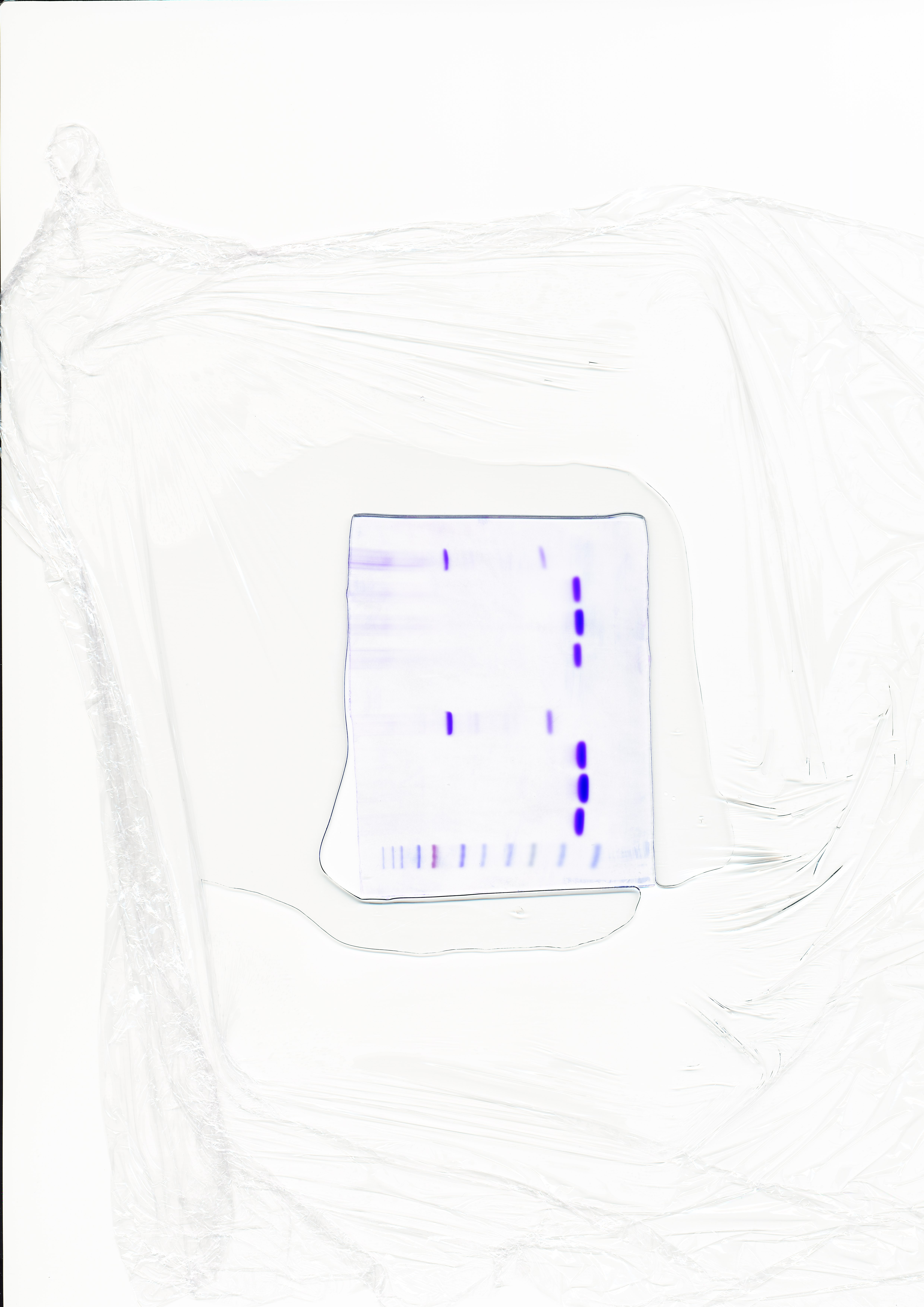

Supplement: Source data 1. [file elife-68164-supp3.zip › Fig1_Sup1Eand1I_Coomassie_Image.jpg]

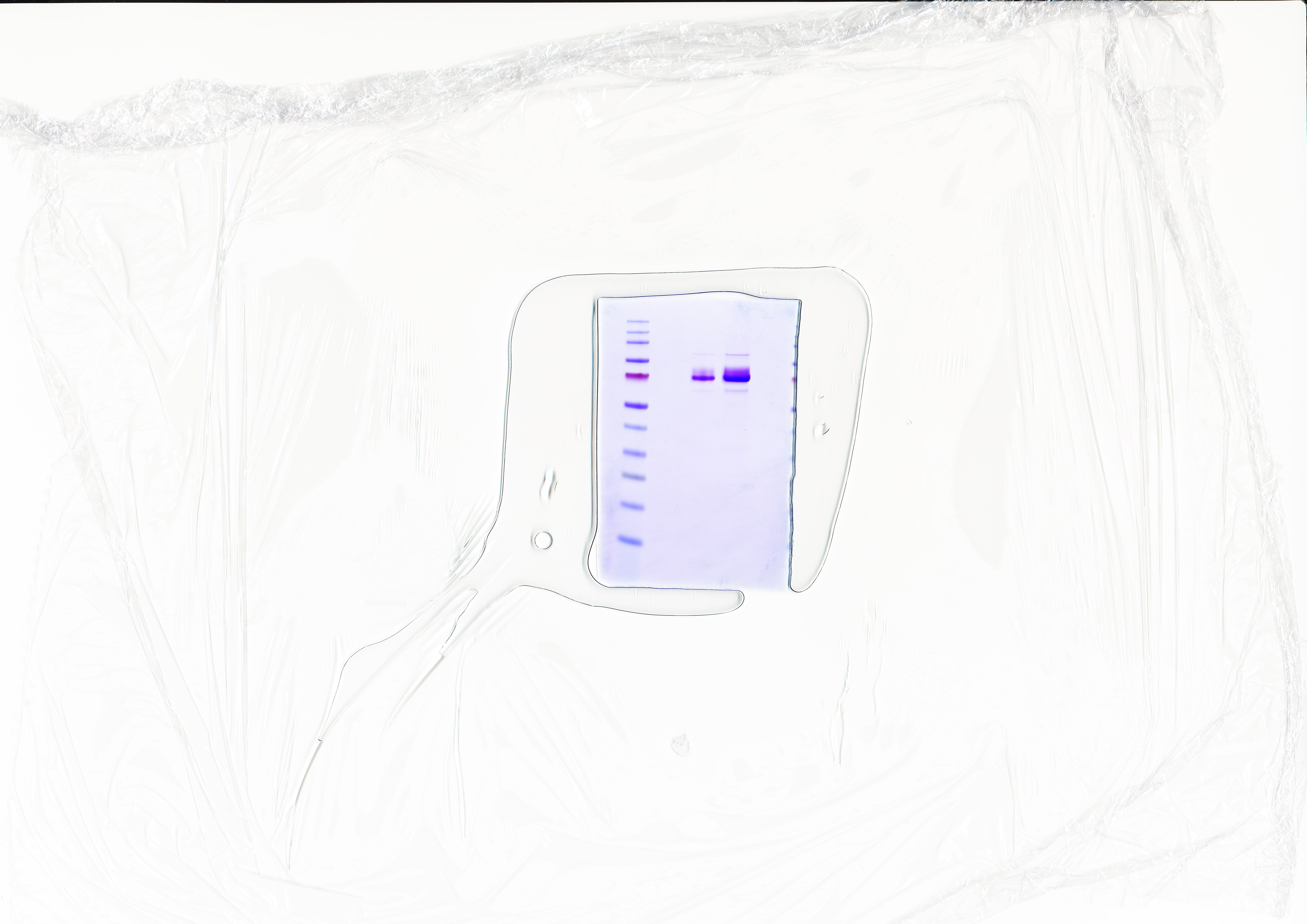

Supplement: Source data 1. [file elife-68164-supp3.zip › Fig1_Sup1G_Coomassie_Image.jpg]

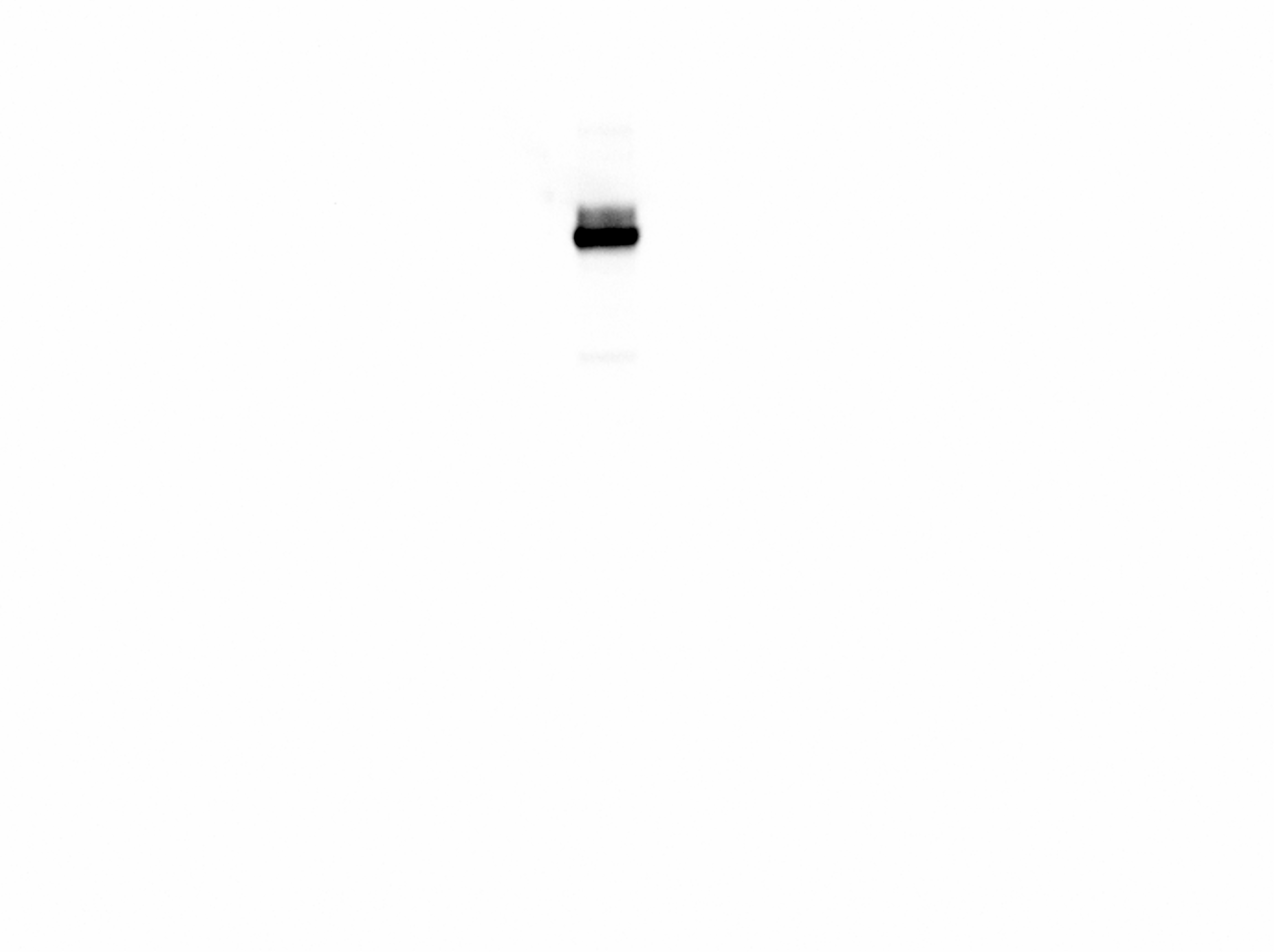

Supplement: Source data 1. [file elife-68164-supp3.zip › Fig1_Sup1G_IB_Original_Image.tif]

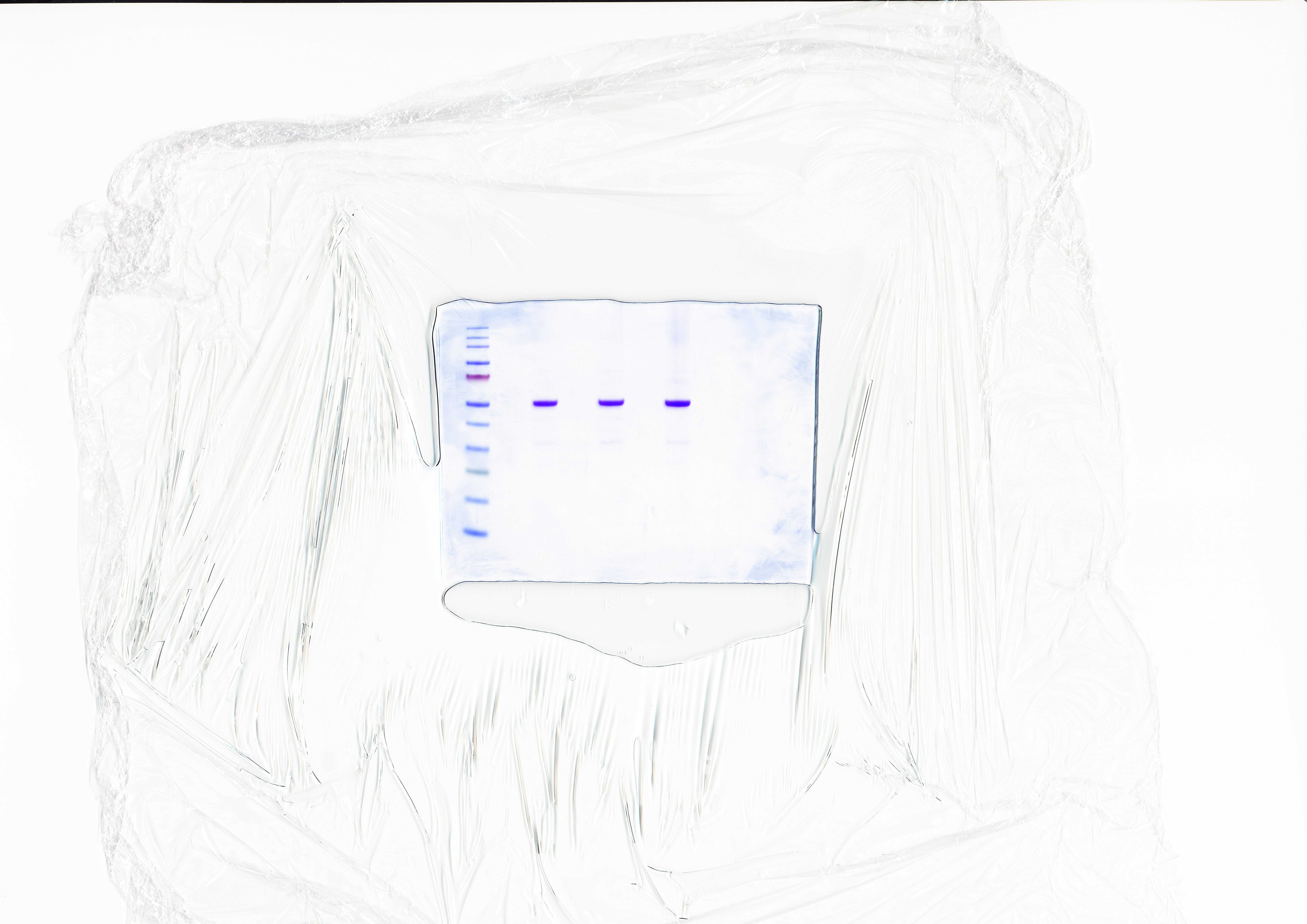

Supplement: Source data 1. [file elife-68164-supp3.zip › Fig1_Sup1H_Coomassie_Original_Image.jpg]

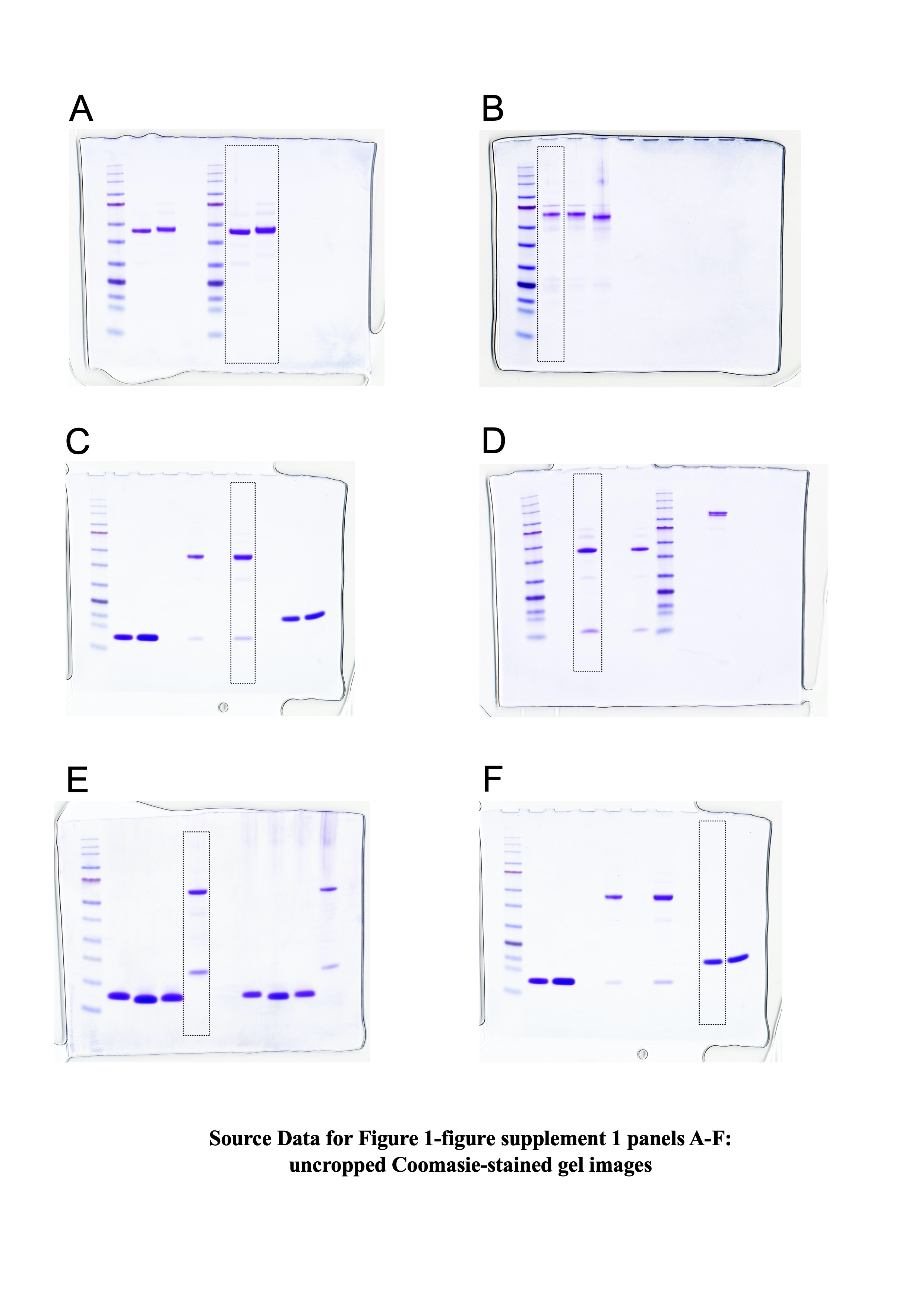

Supplement: Source data 1. [file elife-68164-supp3.zip › Fig1Sup1_PanelsAtoF_Uncropped_Images.jpg]

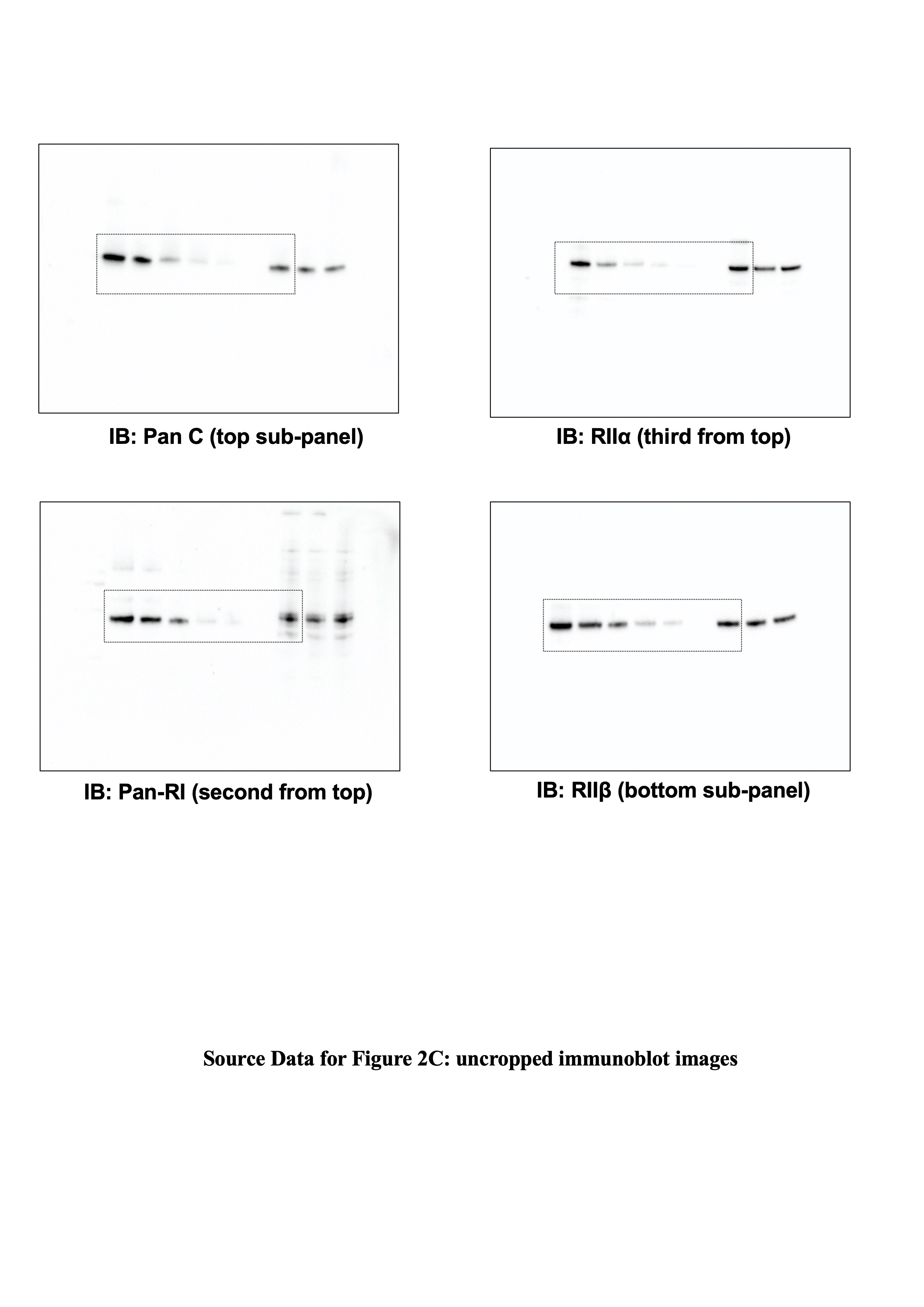

Supplement: Source data 1. [file elife-68164-supp3.zip › Fig2C_Uncropped_Images.jpg]

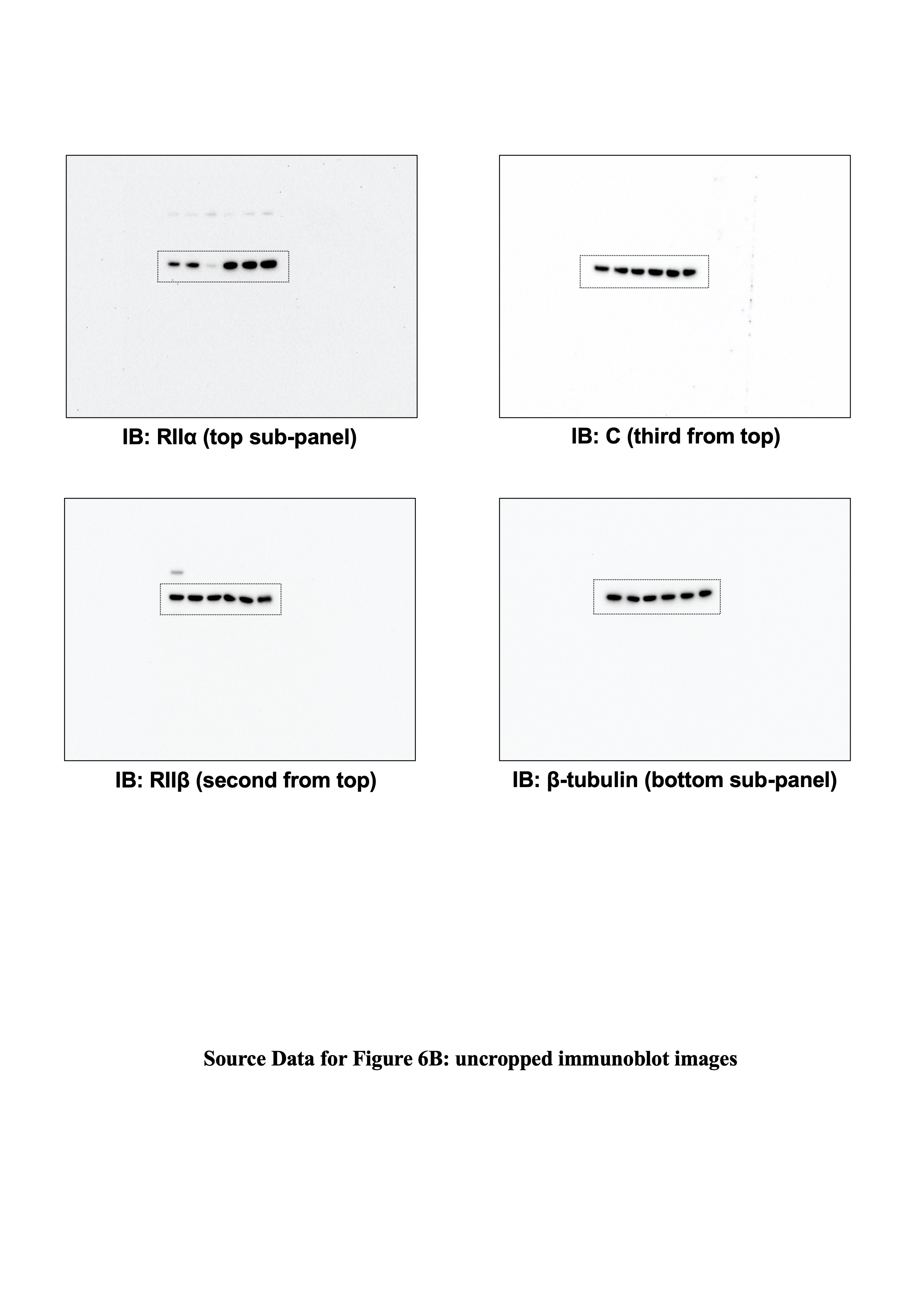

Supplement: Source data 1. [file elife-68164-supp3.zip › Fig6B_Uncropped_Images.jpg]
